# Domestication reshapes the swine gut microbiome: metagenomic insights into taxonomic and functional divergence across wild and domestic populations

**DOI:** 10.3389/fmicb.2026.1854568

**Published:** 2026-07-07

**Authors:** Zhenhua Yan, Fuchen Zhou, Danyang Lin, Donglin Ruan, Yiyi Liu, Ming Yang, Fanming Meng, Sixiu Huang, Langqing Liu, Enqin Zheng, Gengyuan Cai, Jie Yang, Zebin Zhang

**Affiliations:** 1 State Key Laboratory of Swine and Poultry Breeding Industry, Guangdong Laboratory for Lingnan Modern Agriculture, National Engineering Research Center for Breeding Swine Industry, National and Regional Livestock Genebank, Guangdong Gene Bank of Livestock and Poultry, College of Animal Science, South China Agricultural University, Guangzhou, Guangdong, China; 2 College of Animal Science and Technology, Zhongkai University of Agriculture and Engineering, Guangzhou, Guangdong, China; 3 Institute of Animal Science, Guangdong Academy of Agricultural Sciences, Guangdong Key Laboratory of Animal Breeding and Nutrition, Guangzhou, Guangdong, China; 4 Guangdong Provincial Key Laboratory of Agro-animal Genomics and Molecular Breeding, South China Agricultural University, Guangzhou, Guangdong, China; 5 Guangdong Zhongxin Breeding Technology Co., Ltd, Guangzhou, Guangdong, China; 6 Wens Foodstuff Group Co., Ltd., Yunfu Subcenter of Guangdong Laboratory for Lingnan Modern Agriculture, Yunfu, Guangdong, China

**Keywords:** domestic, gut microbiome, metagenomics, microbial diversity, pig

## Abstract

**Background:**

The gut microbiota constitutes a highly diverse, complex, and dynamically evolving ecosystem within the host. However, the domestication process may alter microbial community composition and function. Here, we investigate these shifts using metagenomic analysis.

**Methods:**

Microbial diversity was evaluated using alpha and beta-diversity analysis. Furthermore, LEfSe and Functional analyses were employed to delineate significant disparities in microbial abundance and functional potential between wild boars (WB), Chinese domestic pigs (CDP), and Western domestic pigs (WDP).

**Results:**

Our analysis revealed distinct microbial signatures across populations. WB exhibit greater diversity differentiation from WDP, while showing higher similarity to CDP. WB were significantly enriched in the genera *Treponema, Oscillibacter*, and *Pseudoflavonifractor*. In contrast, Chinese domestic breeds were characterized by *Lactobacillus, Prevotella* and *Ruminococcus*, while WDP retained high abundances of *Alistipes, Bacteroides* and *Clostridium*. Functionally, the wild boar microbiome showed significantly higher activity in pathways related to plant secondary metabolite degradation and nutrient biosynthesis. Conversely, domestic pig microbiomes showed significant enrichment in antimicrobial resistance genes and DNA damage repair pathways.

**Conclusions:**

These findings indicate that domestication has influenced the swine gut microbiota, contributing to distinct compositional and functional divergences. Future research may explore the potential of reintroducing wild-derived probiotics to enhance domestic pig health.

## 1 Introduction

As the “second genome” of animals, the gut microbiota reflects the intricate symbiosis and co-evolution between host and microbe. During thousands of years of domestication, this “second genome” has been substantially reconstituted in response to dramatic changes in the environment, diet, and host genetics (Simon et al., 2019). The gastrointestinal tract harbors trillions of microorganisms spanning the three domains of life—*Bacteria, Archaea*, and *Eukaryota*—alongside viruses. *Bacteria* and *Archaea* constitute over 99% of the total microbial biomass and have consequently garnered the most research attention (Sender et al., 2016; Wampach et al., 2017). Although protozoa represent a minor fraction of the microbiome and exhibit significant variability in relative abundance, their emerging roles as critical ecosystem members are increasingly recognized (Huseyin et al., 2017; Laforest-Lapointe and Arrieta, 2018). In the porcine gut, the microbiota is dominated by anaerobes and facultative anaerobes, with *Bacillota* and *Bacteroidetes* collectively comprising more than 90% of the community. These microbial populations play pivotal roles in modulating immune function, maintaining host health, and facilitating nutrient metabolism and absorption (Donaldson et al., 2016).

The gut microbiota forms an intimate symbiotic relationship with the host, interacting through complex mechanisms to constitute an interdependent and mutually beneficial holobiont (Hooper et al., 1998). These microorganisms orchestrate a wide array of physiological processes, ranging from dietary digestion and nutrient metabolism to immune modulation and environmental adaptation, thereby playing a pivotal role in maintaining host health (Gill et al., 2006). Under normal physiological conditions, a dynamic homeostasis is maintained among the microbiota, the host, and the external environment; this self-regulating system is fundamental to the physiological stability of the host.

Domestication is the process by which the domestic pig has adapted from its wild ancestor, the wild boar, to an intensive farming environment (Kuthyar et al., 2023; Reese et al., 2021). This transition is accompanied by dietary simplification, strict environmental control, and frequent antibiotic application (Ushida et al., 2016; McKenzie et al., 2017), leading to adaptive reshaping of both the host genome and the gut microbiome. In the wild, boars mainly consume high-fiber foods, such as acorns, wild fruits, and roots. Research indicates that such a natural diet may enhance their environmental adaptability and disease resistance (Montes-Sánchez et al., 2020). In contrast, domestic pigs are often fed simplified, high-carbohydrate diets, which studies indicate may lead to reduced disease resistance, changes in gut morphology, and faster growth rates (Ding et al., 2023; Dou et al., 2023).

In this study, we leveraged metagenomic data from 198 pigs spanning 16 diverse breeds to characterize the evolutionary trajectory of the porcine gut microbiome during domestication. Through integrated analyses of taxonomic composition, alpha and beta diversity, LEfSe and functional analyses, we demonstrate significant structural shifts in the microbial landscape and identify key biomarkers associated with the domestication process. By elucidating the impact of domestication on host-microbe interactions, our findings provide critical insights into how dietary transitions have shaped the evolution and functional capacity of the gut microbiota.

## 2 Materials and methods

### 2.1 Animals and sample collection

In this study, we analyzed a comprehensive dataset of 198 porcine metagenomes, comprising 166 datasets retrieved from public repositories and 32 sequenced in-house. The cohort included wild boars, Chinese domestic pigs, and Western domestic breeds. Specifically, the Chinese domestic pigs spanned four geographical regions: Southwest (Tibetan, Congjiang Xiang, Dahe Black, and Diannan Small-ear pigs), South China (Bama Xiang, Huanjiang Xiang, Lantang, and Liangguang Small-spotted pigs), Central China (Ningxiang and Shaziling pigs), and North China (Laiwu and Licha pigs). The Western group consisted of Large White, Landrace, and Duroc breeds. Detailed sample information is provided in Supplementary Table 1.

### 2.2 Data preprocessing

Raw Illumina paired-end reads were first subjected to quality control using fastp (Chen, 2025) (-W 4, -M 20,-n 5,-c -l 50, -w 3)to remove adapters and low-quality sequences. To eliminate host contamination, the clean reads were aligned to the pig reference genome (*Sus scrofa 11.1*) using KneadData (McIver et al., 2018) (–bowtie2-options ‘–very-sensitive –dovetail'), and matching host sequences were discarded to obtain high-quality microbial reads. Subsequently, a two-step co-assembly strategy was employed. First, the clean microbial reads were co-assembled using MEGAHIT (v1.2.9) (Li et al., 2015) with a multi-k-mer strategy (-k 21,29,39,59,79,99,119,141) to generate an initial set of contigs. To capture unassembled genetic information, reads from each sample were mapped back to these initial contigs using Bowtie 2 (v2.4.4) (Langmead and Salzberg, 2012). Unmapped reads were then pooled and subjected to a second round of co-assembly using MEGAHIT. Finally, contigs from both assembly rounds were merged for downstream analysis. We adopted a two-step co-assembly strategy rather than per-sample individual assembly for the following reasons: (i) co-assembly increases the recovery of low-abundance microbial genomes by leveraging read coverage across multiple samples; (ii) it reduces the proportion of fragmented contigs and improves the completeness of gene catalogs; (iii) the second assembly round specifically targets reads that did not map to the initial contigs, capturing strain-variable regions and rare species that would otherwise be lost. This approach is widely recommended for large-scale metagenomic studies where inter-sample comparability and gene discovery are priorities.

Contigs were predicted from the assembled contigs using Prodigal (Hyatt et al., 2010) (-p meta, -f gff) with default parameters to retrieve both nucleotide and protein sequences. A non-redundant gene catalog was constructed using CD-HIT (v4.8.1) (Li and Godzik, 2006) (-as 0.9, -c 0.95,-G 0,-g 0,-T 0,-M 0) by clustering sequences at 95% identity and 90% coverage. Based on this catalog, taxonomic and functional annotations were performed using DIAMOND (Buchfink et al., 2015). For taxonomic classification, non-redundant protein sequences were aligned against the NCBI NR database, retaining the best hits with an *e-value* < 1 × 10^−5^. To ensure high-confidence taxonomic profiling and eliminate background noise, a stringent Best-Hit filtering strategy was applied. For genes characterized by multiple alignment alignments, only the top-scoring alignment with the highest bit score and lowest *e-value* was retained for further analysis. Functional annotation was conducted by aligning the same protein set against the Evolutionary Genealogy of Genes: Non-supervised Orthologous Groups (eggNOG) (v5.0) and Kyoto Encyclopedia of Genes and Genomes (KEGG) databases, using the same *e-value* threshold. Gene abundance profiles were quantified using Salmon (v1.5.1) (Patro et al., 2017) by indexing the non-redundant nucleotide sequences. We applied the MMUPHin package to correct for batch effects in the species abundance matrix derived from multiple study sources, followed by PERMANOVA analysis, which yielded an *R*^2^ of 0.02 and a *P-value* > 0.05. In total, 820 families, 4,079 genus, and 41,896 species were identified in this study.

### 2.3 Statistical analysis

#### 2.3.1 Species composition, and alpha and beta diversity analyses

Taxonomic abundances were aggregated at the family, genus, and species levels, with relative abundances calculated based on the mean values within each group (CDP, WB, and WDP). The top 10 most abundant taxa were identified, while all remaining taxa were grouped as “Others.”

Alpha and beta diversity analyses were performed using R (v4.2.3) with the vegan package. Community richness and evenness (alpha diversity) were assessed via Shannon, Simpson, and Invsimpson indices. Significant differences between groups were determined using the Wilcoxon rank-sum test. For beta diversity, species abundance data were first normalized to relative abundances. Bray-curtis distance matrices were then constructed using the vegdist function. Variations in community structure were visualized through Principal Coordinates Analysis (PCoA), with statistical significance across groups evaluated by Permutational Multivariate Analysis of Variance (PERMANOVA) based on 999 permutations (Anderson and Walsh, 2013).

#### 2.3.2 LEfSe and functional enrichment analyses

We employed the Linear Discriminant Analysis Effect Size method via the LEfSe to identify group-specific biomarkers based on taxonomic abundance profiles A Linear Discriminant Analysis (LDA) score threshold of >3.0 was applied to distinguish characterizing microbial taxa with significant differential abundance across groups.

Functional annotation files derived from the metagenomic data were utilized for downstream analysis. To account for variations in sequencing depth, raw abundances of metabolic pathways were normalized to relative abundances (%). Differential functional profiles between groups were identified using the Wilcoxon rank-sum test. To control for multiple hypothesis testing, *P-values* were adjusted using the Benjamini-Hochberg method to calculate the false discovery rate (FDR), with a threshold of *P-adj* < 0.05 considered statistically significant. Finally, extended bar plots were generated in R to represent the mean relative abundance of these significant pathways alongside the 95% confidence intervals (CIs) for the mean difference between groups.

## 3 Results

### 3.1 Microbial composition analysis of wild boar, Chinese domestic pig, and Western domestic pig populations

**Figure 1:**
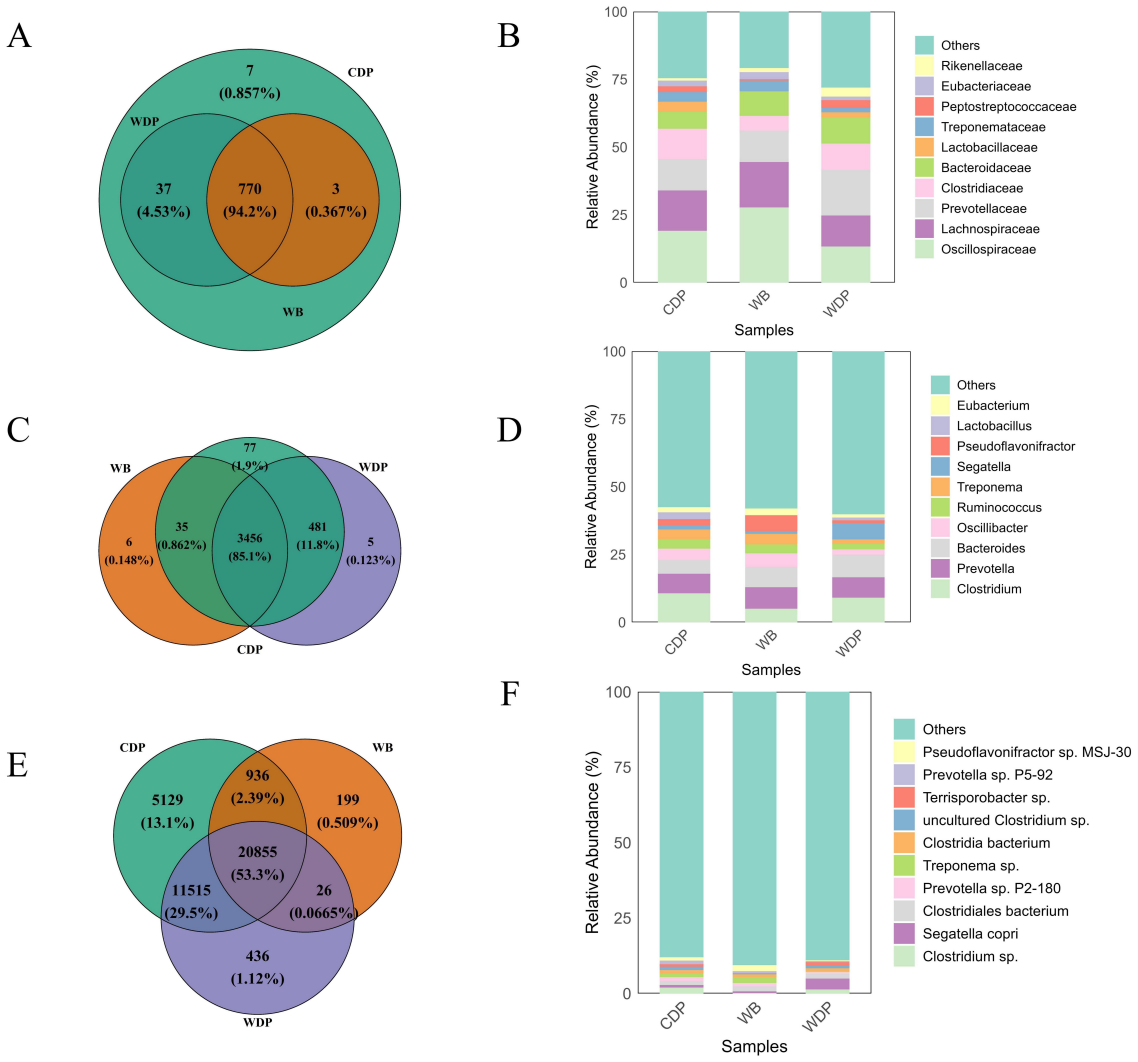
Microbiota composition and shared taxa across CDP, WB, and WDP groups. **(A, B)** Family-level analysis. **(A)** Venn diagram displaying the number of shared and unique bacterial families among the three groups. **(B)** Relative abundance of the top 10 bacterial families, with remaining taxa grouped as “Others.” **(C, D)** Genus-level analysis. **(C)** Venn diagram illustrating the distribution of shared and unique genera across groups. **(D)** Stacked bar plot representing the relative abundance of the top 10 bacterial genera. **(E, F)** Species-level analysis. **(E)** Venn diagram showing the intersection of bacterial species across the experimental groups. **(F)** Relative abundance of the top 10 bacterial species, highlighting the dominant taxonomic shifts.

We characterized the taxonomic distribution of the microbial community at the family, genus, and species levels. For each taxonomic rank, the community overlap among groups and the relative abundances of the top 10 dominant taxa were systematically evaluated and visualized (Figure 1). At the family level, 773, 814, and 807 families were annotated in the WB, CDP, and WDP (Figure 1A). Notably, the microbial families identified in the CDP group encompassed those found in both WB and WDP. Taxonomic abundance analysis further characterized the distribution of dominant taxa across the three groups (Figure 1B). In the WB group, O*scillospiraceae* exhibited the highest relative abundance (27.72%), followed by *Lachnospiraceae* (16.76%) and *Prevotellaceae* (11.56%), while *Lactobacillaceae* (0.17%) and *Peptostreptococcaceae* (0.52%) were present at their lowest levels compared to the other groups. Within the CDP group, the four most prevalent families were *Oscillospiraceae* (19.10%), *Lachnospiraceae* (14.88%), *Prevotellaceae* (11.56%), and *Clostridiaceae* (11.22%). In contrast, the WDP community was dominated by *Prevotellaceae* (16.76%), followed by *Oscillospiraceae* (13.29%), *Lachnospiraceae* (11.48%), and *Clostridiaceae* (9.77%), with *Rikenellaceae* (3.31%) being significantly more abundant than in the other two groups.

At the genus level, 3,497, 3,568, and 3,498 genera were identified in the WB, CDP, and WDP groups (Figure 1C), respectively, with the CDP group exhibiting the broadest taxonomic coverage. Taxonomic profiling further elucidated the distribution of dominant genera across the three cohorts (Figure 1D). In the WB group, *Prevotella* exhibited the highest relative abundance (7.96%), followed by *Bacteroides* (7.45%) and *Pseudoflavonifractor* (5.86%), while *Lactobacillus* (0.05%) and *Segatella* (0.98%) were present at their lowest levels compared to the other groups. Within the CDP group, the four most prevalent genera were *Clostridium* (10.72%), *Prevotella* (7.19%), *Bacteroides* (5.02%), and *Oscillibacter* (4.24%). In contrast, the WDP community was dominated by *Clostridium* (9.06%), followed by *Bacteroides* (8.30%), *Prevotella* (7.54%), and *Segatella* (6.00%), with *Segatella* being significantly more abundant than in the other two groups (CDP: 1.52%; WB: 0.98%).

At the species level, a total of 41,896 species were identified across the three groups, with 20,855 core species shared among all samples, accounting for 53.3% of the total. The CDP group exhibited the highest taxonomic richness, harboring 5,129 unique species (13.1%). Notably, the overlap between CDP and WDP was substantially higher (11,515 species, 29.5%) than that between CDP and WB (936 species, 2.39%) (Figure 1E). Regarding relative abundance, the top 10 species displayed marked heterogeneity across groups (Figure 1F). In the WB group, *Pseudoflavonifractor sp. MSJ-30* (1.99%) and *Treponema sp*. (1.96%) were the most prevalent, whereas the levels of *Clostridium sp*. (0.16%) and *Terrisporobacter sp*. (0.06%) were significantly lower than those in the domestic pig groups. The dominant species in the CDP group primarily included *Clostridium sp*. (1.99%), *Clostridiales bacterium* (1.30%), and *Prevotella sp. P2-180* (1.30%). Of particular interest, *Segatella copri* exhibited a striking dominance in the WDP group (3.61%), far exceeding its proportions in CDP (0.81%) and WB (0.56%); similarly, *Terrisporobacter sp*. (1.17%) was also markedly enriched in the WDP group.

### 3.2 Alpha and beta diversity of microbial communities in wild boar, Chinese domestic pigs, and Western domestic pigs

**Figure 2:**
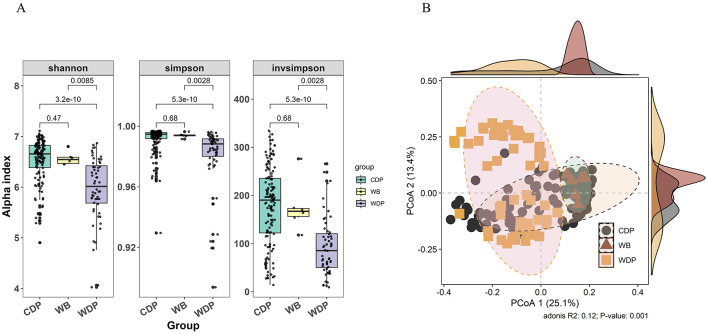
Diversity analysis of the microbial community among groups. **(A)** Alpha diversity indices. Box plots representing the Shannon, Simpson, and inverse Simpson indices across the CDP, WB, and WDP groups. Each point denotes an individual sample. Statistical significance was determined using Wilcoxon rank-sum test, with *P-values* indicated above the brackets. **(B)** Beta diversity analysis. Principal Coordinate Analysis plot based on Bray-Curtis distances. The percentages in parentheses on the axes (e.g., PCo1 (21.4%)) indicate the proportion of total variance explained by each principal coordinate. Each point represents a sample, colored by group (CDP, WB, WDP). Marginal density plots show the distribution along each axis. PERMANOVA results (R^2^ = 0.11, P = 0.001) indicate significant separation among groups.

The Shannon, Simpson, and inverseimpson indices provide complementary assessments of alpha diversity, capturing richness, dominance weighting, and effective number of species, respectively. Alpha diversity analysis (Figure 2A) revealed a high degree of similarity in community richness and evenness between CDP and WB, with no significant differences observed in Shannon (*P* = 0.47), Simpson (*P* = 0.68), or Invsimpson (*P* = 0.68) indices. In contrast, the alpha diversity of WDP was significantly lower than that of the other two groups, with Shannon index values being markedly reduced compared to both CDP (*P* = 3.2 10^−10^) and WB (P = 0.0085). Similar significant declines were observed for the Simpson and Inverse Simpson indices. Regarding beta diversity (Figure 2B), PCoA based on Bray-Curtis distances demonstrated declustering of the three groups, indicating substantial shifts in microbial community composition. PERMANOVA further confirmed that group identity explained 11% of the variation in gut microbiota structure (R^2^ = 0.11, *P* = 0.001). Collectively, these results suggest that while Chinese domestic pigs have maintained a microbial diversity level comparable to that of wild boars during domestication, the intensive selection and distinct genetic background of Western domestic pigs have significantly altered and reduced the complexity and homeostatic structure of their gut microbiota.

### 3.3 Identification of microbial biomarkers across different groups

#### 3.3.1 Identification of microbial biomarkers differentiating wild and domestic pigs

**Figure 3:**
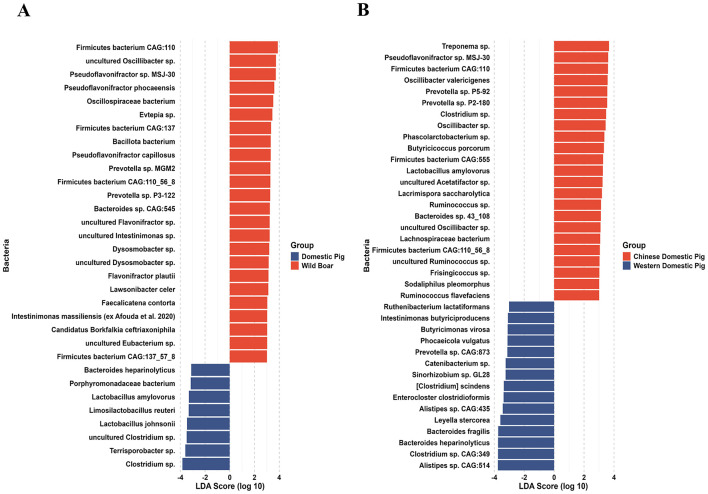
Identification of differentially abundant bacterial taxa between Wild Boars and Domestic Pigs using LEfSe analysis. **(A)** Histogram of the LDA scores for differentially abundant taxa between Domestic Pigs (blue) and Wild Boars (red). **(B)** Histogram of the LDA scores for differentially abundant taxa between Chinese Domestic Pigs (red) and Western Domestic Pigs (blue).

To identify statistically significant microbial biomarkers distinguishing domestic pigs from wild boars, LEfSe analysis was performed using a threshold of LDA score >3.0 (Figure 3A). In the wild boar group, 24 enriched taxa were identified; the most substantial contributors included *Firmicutes bacterium CAG:110, uncultured Oscillibacter sp., Pseudoflavonifractor sp. MSJ-30*, and *Pseudoflavonifractor phocaeensis*. Additionally, *Oscillospiraceae bacterium* and several *Prevotella* strains were significantly enriched in this group. However, the domestic pig group exhibited 8 characteristic species, with *Clostridium sp*. displaying the highest discriminative power, followed by *Terrisporobacter sp*. and *uncultured Clostridium sp*. Notably, three *Lactobacillus* species—*Lactobacillus johnsonii, Limosilactobacillus reuteri*, and *Lactobacillus amylovorus*—were identified as key biomarkers in domestic pigs. These differential biomarkers not only highlight the distinct gut environments of domestic and wild pigs but also reflect the profound impact of domestication pressure on the evolution of the porcine core microbiota.

#### 3.3.2 Identification of gut microbial biomarkers in Chinese and Western domestic pigs

To screen for biomarkers differentiating CDP from WDP, LEfSe analysis was conducted with a strict LDA score threshold of 3.0. In the CDP group, 21 taxa were identified as significantly enriched (Figure 3B). Among these, *Treponema sp., Pseudoflavonifractor sp. MSJ-30, Firmicutes bacterium CAG:110*, and *Oscillibacter valericigenes* exhibited the highest discriminatory power. Notably, *Lactobacillus amylovorus* also showed marked enrichment within the CDP cohort. In contrast, the WDP group was characterized by the enrichment of 15 signature species, with *Alistipes sp. CAG:514* demonstrating the strongest discriminative potential, followed closely by *Clostridium sp. CAG:349, Bacteroides heparinolyticus*, and *Bacteroides fragilis*. The identification of these differential biomarkers not only delineates the unique gut microbial landscapes of Chinese and Western breeds but also reveals significant divergence in the evolutionary trajectories of their dominant microbiota.

### 3.4 Functional analysis of gut metagenomes across different group

#### 3.4.1 Functional disparities between wild boars and domestic pigs

**Figure 4:**
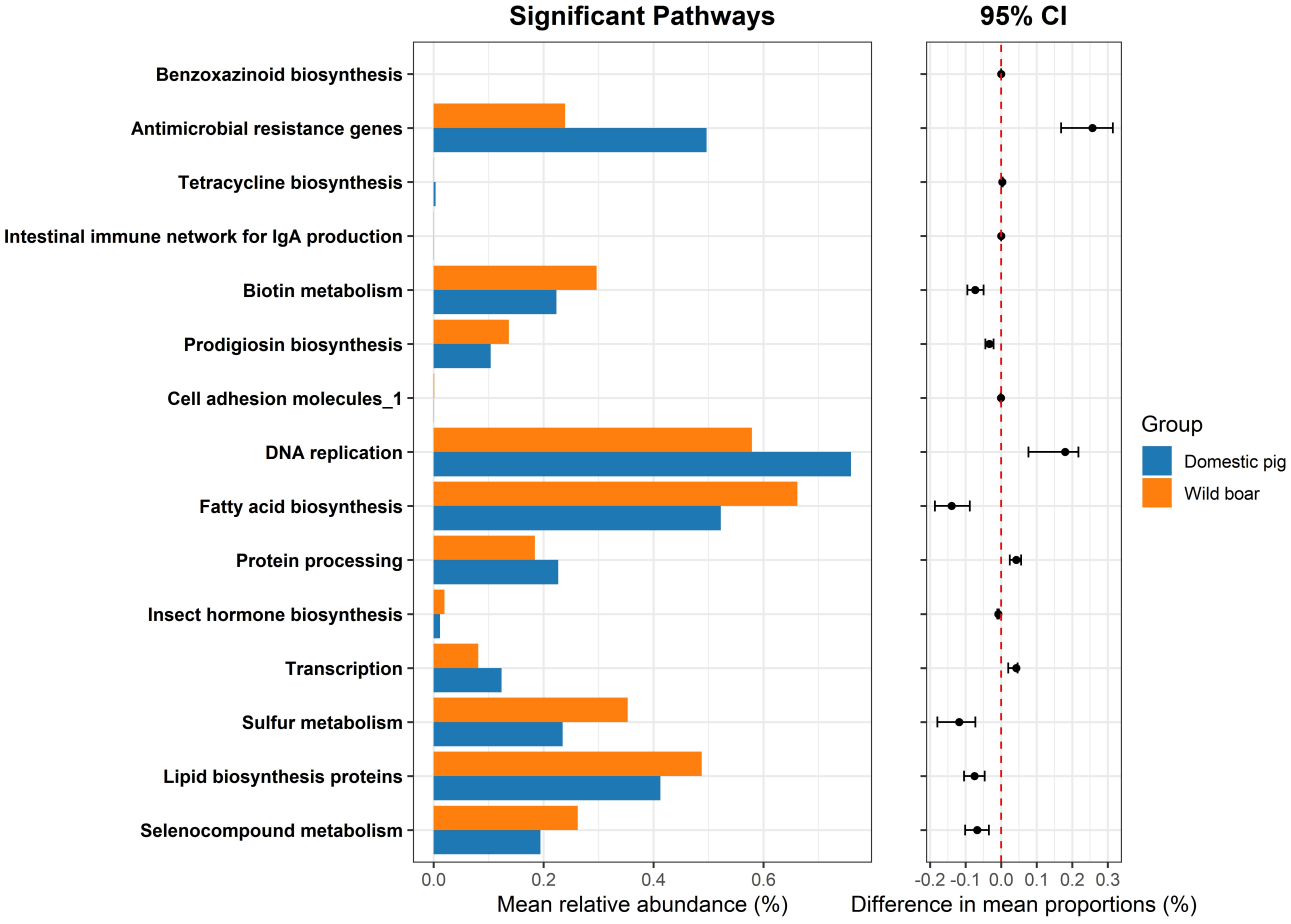
Top 15 functional pathways with significant differences between domestic pigs and wild boars identified by Functional analysis. **Left**: Bar plot representing the mean relative abundance (%) of each pathway. Blue bars represent Domestic pigs, and orange bars represent Wild boars. **Right**: Dot plot showing the difference in mean proportions (%) between the two groups with 95% CI. Points to the right of zero indicate enrichment in Domestic pigs, while points to the left indicate enrichment in Wild boars.

Metagenomic functional analysis revealed a marked divergence in survival strategies and metabolic patterns between the gut microbiomes of domestic pigs and wild boars. This study visualized the top 15 pathways (Figure 4, See Supplementary Table 2 for details). The gut microbiota of domestic pigs exhibited distinct adaptations to the intensive farming environment. The most prominent feature of this group was the profound enrichment of resistance and defense mechanisms. This included antimicrobial resistance genes and various pathways associated with the maintenance of genomic stability, such as DNA replication, DNA repair and recombination proteins, and Mismatch repair. Furthermore, elevated activity was observed in the biosynthesis of specific secondary metabolites (e.g., Tetracycline biosynthesis, novo biocin biosynthesis) and Nucleotide metabolism. These patterns likely reflect a core requirement for maintaining microbial survival and genetic fidelity under conditions of high-intensity anthropogenic intervention.

Conversely, the microbiome of wild boars displayed greater metabolic plasticity regarding nutrient acquisition and adaptation to natural environments. This group was significantly enriched in broad biosynthetic and basal metabolic pathways, including those for essential amino acids (e.g., Arginine and proline metabolism, Cysteine and methionine metabolism, Phenylalanine, tyrosine and tryptophan biosynthesis) and vitamin/cofactor metabolism (e.g., Biotin metabolism, Vitamin B6 metabolism). Concurrently, the wild boar group showed heightened activity in complex carbon source utilization and energy acquisition (e.g., Carbon fixation pathways in prokaryotes, Fatty acid biosynthesis) and lipid metabolism (Lipid biosynthesis proteins). Notably, pathways involved in the synthesis and degradation of plant secondary metabolites (e.g., Glucosinolate biosynthesis, Limonene and pinene degradation, Benzoxazinoid biosynthesis) were significantly enriched, aligning closely with the complex omnivorous diet of wild boars and the necessity to degrade natural plant defense chemicals.

#### 3.4.2 Functional divergence between western domestic and Chinese domestic pigs

**Figure 5:**
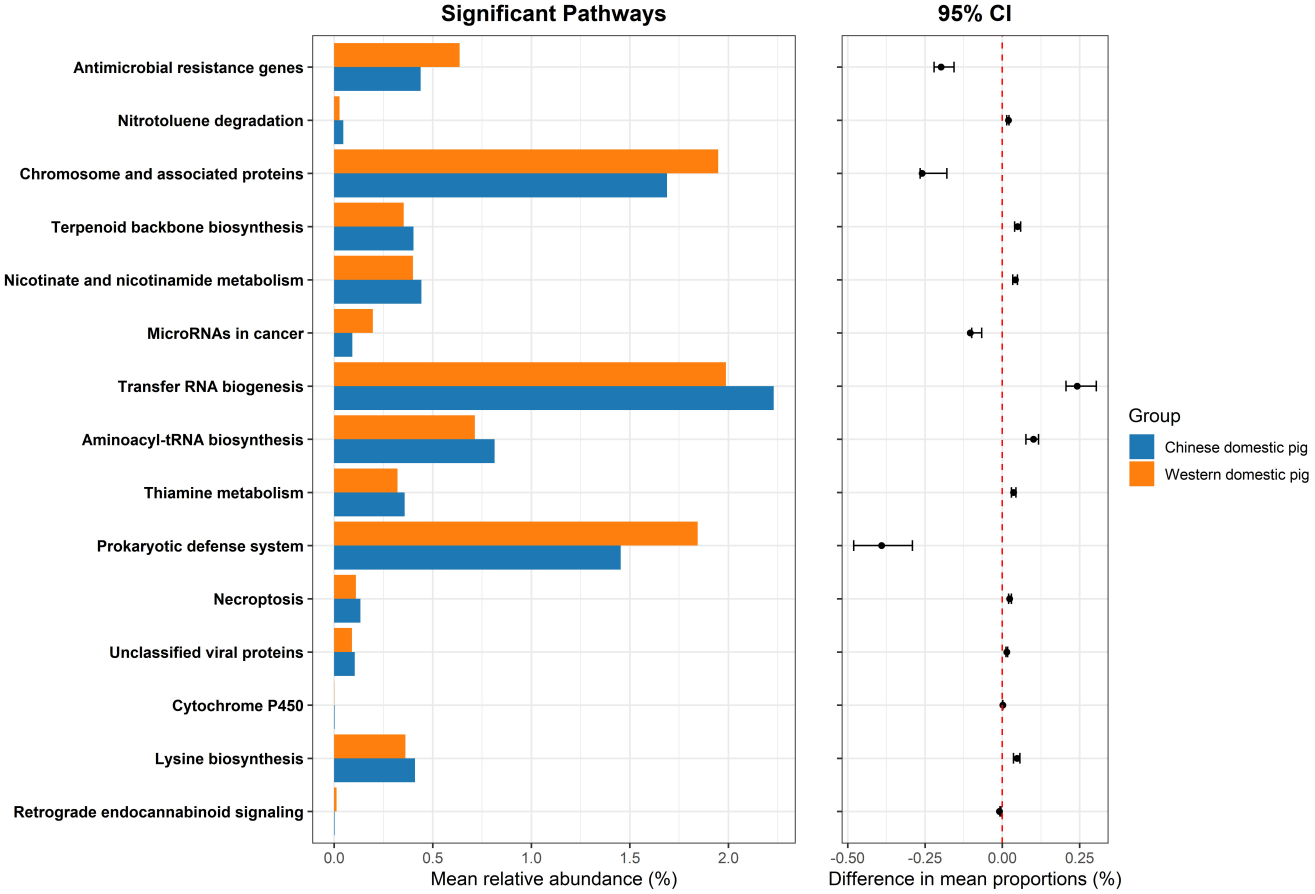
Top 15 functional pathways with significant differences between Chinese and Western domestic pigs identified by Functional analysis. **Left**: Bar plot representing the mean relative abundance (%) of each pathway. Blue bars represent Chinese domestic pigs, and orange bars represent Western domestic pigs. **Right**: Dot plot showing the difference in mean proportions (%) between the two groups with 95% CI.

Metagenomic functional analysis further elucidated significant functional differentiation in survival strategies and metabolic modes between Western domestic and Chinese domestic pigs. This study visualized the top 15 pathways (Figure 5, Supplementary Table 3 for details). The gut microbiota of Western domestic pigs was characterized by enhanced resistance and the maintenance of genetic stability. A core feature of this group was the extensive enrichment of pathways related to defense mechanisms and genomic fidelity. Specifically, Prokaryotic defense systems and multiple antimicrobial resistance pathways (e.g., Antimicrobial resistance genes, Cationic antimicrobial peptide [CAMP] resistance) were highly active. Simultaneously, pathways related to genomic stability, including DNA replication proteins, DNA repair and recombination proteins, Mismatch repair, and Chromosome and associated proteins, were significantly enriched. Moreover, this group showed a significant enrichment in Oxidative phosphorylation, a core energy metabolic pathway. This suggests a survival strategy centered on reinforcing defense/repair mechanisms and maximizing energy efficiency to maintain population stability under the stress of intensive farming.

In contrast, the microbiota of Chinese domestic pigs exhibited higher activity in growth and development, nutritional plasticity, and environmental sensing. The most striking feature was the significant enrichment of pathways related to protein synthesis and processing, including Transfer RNA biogenesis, Ribosome biogenesis, and Aminoacyl-tRNA biosynthesis. In terms of nutrient metabolism, the Chinese group displayed broad biosynthetic advantages, with significant enrichment in essential amino acid metabolism (e.g., Alanine, aspartate and glutamate metabolism; Leucine and isoleucine biosynthesis; Tyrosine and tryptophan biosynthesis) and carbohydrate utilization (e.g., Starch and sucrose metabolism, Pyruvate metabolism). Notably, pathways involved in environmental information processing (Two-component system, Quorum sensing) and bacterial motility (Bacterial motility proteins, Flagellar assembly) were more active in this group. This implies a greater capacity for active colonization, resource competition, and metabolic regulation within complex habitats, reflecting an adaptive divergence associated with traditional farming practices or specific dietary components.

## 4 Discussion

Gut microbial diversity is widely recognized as a pivotal indicator of host health and ecosystem stability. In this study, alpha diversity was significantly lower in WDP compared to CDP and WB, whereas CDP and WB exhibited highly comparable diversity profiles. This finding concurs with previous hypotheses suggesting that animal domestication and industrialization are associated with a “loss of diversity” in the microbiota (Wei et al., 2022; Bae et al., 2025). The diminished diversity observed in WDP may be attributed to highly standardized commercial diets, intensive selection pressure, and the prophylactic use of antibiotics in industrial farming, all of which significantly contract the available ecological niches (Alauzet et al., 2023). Conversely, although CDP have also undergone domestication, they maintain a community richness similar to that of wild boars, likely due to traditional husbandry practices involving high-fiber intake, diversified feed sources, and a distinct genetic background (Stanton and Canale-Parola, 1980). The distinct clustering revealed by PCoA further underscores that domestication serves as a primary driver in the evolutionary shaping of the porcine gut microbiota (Varel, 1987; Watanabe et al., 2012).

The high abundance of *Oscillospiraceae* and *Oscillibacter* in WB is closely linked to their wild foraging behavior. These taxa are widely recognized for their role in degrading complex carbohydrates, such as cellulose and hemicellulose, thereby facilitating energy extraction in natural environments with fluctuating food resources (Iino et al., 2007; Ma et al., 2022). Notably, CDP exhibited a significant enrichment of *Lactobacillus* species, specifically *L. johnsonii* and *L. amylovorus*. As established probiotics, *Lactobacillus* plays a pivotal role in maintaining the intestinal barrier and conferring pathogen resistance; its enrichment in domestic pigs may be induced by high-starch diets or result from long-term artificial selection for disease-resistant phenotypes. In WDP, *Segatella copri* displayed a striking dominance. While *S. copri* is typically associated with plant polysaccharide degradation, its overproliferation under high-energy dietary regimes has been linked to pro-inflammatory responses in the host (Carlier et al., 2010; Li et al., 2024). This suggests potential gut health risks in Western domestic pigs under the physiological strain of rapid growth and high metabolic demand.

Functional profiling in this study revealed a distinct divergence in metabolic strategies between wild boars and domestic pigs. The microbiota of WB was significantly enriched in pathways related to the biosynthesis of vitamins (e.g., B6 and biotin), essential amino acid metabolism, and the degradation of plant secondary metabolites, such as glucosinolates and terpenoids. These findings reflect the reliance of omnivorous wild boars on their gut microbiota to synthesize limiting nutrients and detoxify defensive plant toxins, thereby enhancing their fitness in unpredictable natural environments (Braune and Blaut, 2016; Liu et al., 2021). In stark contrast, the microbiota of domestic pigs (WDP and CDP) exhibited pronounced “intensification signatures.” The enrichment of antibiotic resistance genes and DNA repair mechanisms likely reflects the sustained antibiotic pressure and genomic instability associated with rapid cell proliferation in intensive production environments (Pu et al., 2025).

In the comparison between CDP and WDP, Chinese domestic pigs exhibited a significantly higher relative abundance of pathways involved in protein translation (notably tRNA and ribosome biogenesis) and amino acid biosynthesis. This active protein metabolism may be linked to the superior environmental resilience of indigenous Chinese breeds and their high efficiency in utilizing low-protein diets (He et al., 2019). Conversely, the WDP microbiota was significantly enriched in oxidative phosphorylation pathways, suggesting that these microbial communities may support the rapid growth rates of their hosts through enhanced energy production (Cheng et al., 2018). Furthermore, the enrichment of prokaryotic defense systems (e.g., CRISPR-Cas and restriction-modification systems) in WDP likely reflects an adaptive co-evolution of the microbiota in response to frequent bacteriophage infections and exogenous DNA incursions common in closed, high-density farming environments (Millman et al., 2022).

## 5 Conclusions

Through metagenomic sequencing, we conducted a comparative analysis of the gut microbiota between wild boars and domestic pigs, highlighting the profound shifts associated with domestication. Our results demonstrate that the domestication process has fundamentally reconfigured the taxonomic landscape of the porcine gut, resulting in distinct signatures in both species composition and microbial diversity. Specifically, the wild boar microbiota is characterized by a significant enrichment of genera such as *Treponema, Oscillibacter*, and *Pseudoflavonifractor*. In contrast, Chinese indigenous pigs have retained a prevalence of *Lactobacillus, Prevotella*, and *Ruminococcus*, while Western breeds are dominated by *Alistipes, Bacteroides*, and *Clostridium*. Functional profiling further reveals that the wild boar microbiota excels in the degradation of plant secondary metabolites and nutrient biosynthesis. Conversely, the domestic pig microbiota shows a marked enrichment in antimicrobial resistance genes and DNA damage repair pathways. Collectively, our study indicates a divergence in both taxonomic abundance and functional capacity between wild and domestic pigs, pointing to a potential influence of domestication on gut microbial homeostasis. Future research should explore the potential of reintroducing wild-derived probiotic candidates, such as *Pseudoflavonifractor sp. MSJ-30, Oscillibacter valericigenes, and Treponema sp*., which were significantly enriched in wild boars and may confer benefits related to fiber degradation and metabolic flexibility, to enhance domestic pig health.

## Data Availability

The raw sequencing data generated in this study have been deposited in publicly accessible repositories. Specifically, the data are available under the following accession numbers: NCBI BioProject (PRJNA872826, PRJNA1147800, PRJNA1079049, and PRJNA747893; accessible via https://www.ncbi.nlm.nih.gov/bioproject/ with the corresponding accession numbers), China National GeneBank (CNGB) project (CNP0000824 and CNP0002106; accessible via https://db.cngb.org/ with the project accession), and China Read Archive (CRA) accession CRA042002 (accessible via https://db.cngb.org/cra/ with the accession). A complete list of these accession numbers is also provided in Supplementary Table S1.
